# Effects of Gamification on the Benefits of Student Response Systems in Learning of Human Anatomy: Three Experimental Studies

**DOI:** 10.3390/ijerph182413210

**Published:** 2021-12-15

**Authors:** Juan J. López-Jiménez, José L. Fernández-Alemán, José A. García-Berná, Laura López González, Ofelia González Sequeros, Joaquín Nicolás Ros, Juan M. Carrillo de Gea, Ali Idri, Ambrosio Toval

**Affiliations:** 1 Department of Informatics and System, Faculty of Computer Science, University of Murcia, 30100 Murcia, Spain; aleman@um.es (J.L.F.-A.); josealberto.garcia1@um.es (J.A.G.-B.); jnr@um.es (J.N.R.); jmcdg1@um.es (J.M.C.d.G.); atoval@um.es (A.T.); 2 Department of Human Anatomy, Faculty of Medicine, University of Murcia, 30100 Murcia, Spain; laura.lopezgonzalez@um.es (L.L.G.); sequeros@um.es (O.G.S.); 3 Software Project Management Research Team, ENSIAS, Mohammed V University in Rabat, Rabat 10000, Morocco; ali.idri@um6p.ma

**Keywords:** gamified audience response system, E-learning, human anatomy, experiment, badges

## Abstract

This paper presents three experiments to assess the impact of gamifying an audience response system on the perceptions and educational performance of students. An audience response system called SIDRA (Immediate Audience Response System in Spanish) and two audience response systems with gamification features, R-G-SIDRA (gamified SIDRA with ranking) and RB-G-SIDRA (gamified SIDRA with ranking and badges), were used in a General and Descriptive Human Anatomy course. Students participated in an empirical study. In the academic year 2019–2020, a total of 90 students used RB-G-SIDRA, 90 students employed R-G-SIDRA in the academic year 2018–2019, and 92 students used SIDRA in the academic year 2017–2018. Statistically significant differences were found between final exam grades obtained by using RB-G-SIDRA and SIDRA, U = 39.211 adjusted *p* = 0.001 and RB-G-SIDRA and R-G-SIDRA U = 31.157 adjusted *p* = 0.015, thus finding strong evidence with respect to the benefit of the badges used in RB-G-SIDRA. Moreover, in the students’ SIDRA systems scores, statistically significant differences were found between RB-G-SIDRA and SIDRA, U = −90.521 adjusted *p* < 0.001, and between R-G-SIDRA and SIDRA, U = −87.998 adjusted *p* < 0.001. Significant correlations between individual and team scores were also found in all of the tests in RB-G-SIDRA and G-SIDRA. The students expressed satisfaction, engagement, and motivation with SIDRA, R-G-SIDRA, and RB-G-SIDRA, thus obtaining a final average assessment of 4.28, 4.61, and 4.47 out of 5, respectively. Students perform better academically with gamified versus non-gamified audience response systems. Findings can be used to build a gamified adaptive learning system.

## 1. Introduction

Clickers are an interactive learning tool used to ask students questions in class. These tools can be used to assess the academic achievement of students over a short period of time [1]. The first clickers were handheld devices on which students had to answer questions proposed by professors in class. Clickers have evolved into web-based systems [2,3], which allow students to use their smartphone as the handheld device, thus resulting in classroom response systems (CRSs).

Interactive learning activities have shown to improve the learning outcomes. Particularly, there is evidence that CRSs promote conceptual knowledge [4]. Therefore, CRSs are a valuable instrument for education in health sciences and a reliable and objective professor evaluation resource to assess complex capabilities and understanding.

Gamification is associated with the adoption of game mechanics, techniques, and game theory in non-gaming contexts [5,6]. Feedback, challenges, social sharing, rewards, leaderboards (rankings), points, tips, levels, avatars, badges, and user generated content are gamification elements employed successfully in literature [7]. Although a comprehensive list of different types of game elements has been published in grey literature [8], there is a lack of consensus with regards to terminology employed in game elements [9]. For example, different terms are used for rewards: badges, donuts, or iPads.

A large number of studies have used gamification approaches in health professions’ education. However, research is ongoing as to when and for what reasons gamification can be a suitable educational tool [10,11]. Gamification features can be added to CRSs, which leads to increase student concentration and active participation. The game principles have been applied to CRSs such as Kahoot and Socrative to promote fun learning. Gamified CRS sessions are perceived as being more interesting than traditional e-learning quizzes [12].

This paper presents three experiments to evaluate the impact of ranking, badges, teams, and points in a gamified mobile CRS on students’ academic performance and perceptions. To the best of the authors’ knowledge, no other studies have compared different gamification elements used in a CRS. The results of this experiment will help designers and developers to build more effective CRSs in teaching in general, and human anatomy education in particular. As suggested by Ahmad et al. [13], learning techniques used in the teaching of human anatomy must be modernized to take advantage of 21st century technology. Our work adds to the corpus of knowledge of digital learning innovations in the teaching of human anatomy [14,15].

## 2. Related Work

CRSs have been successfully used in pharmacy [16,17], pediatrics [18,19], advanced nursing therapeutic [20], multidisciplinary healthcare providers [21], nursing health assessment [22], medical-surgical [23], family medicine residents [24], ethics [25], anatomy and physiology [26], pathophysiology [27], anticoagulation [28], emergency [29], physical basis of medicine [30], clinical medicine [31], cardiology [32], medical prescription [33], pre-clinical medicine [12], and histology [34]. CRSs can employ many kinds of questions: multiple-choice questions (MCQs), find on image, quiz by combining items, fill in blanks, true/false questions, find a number, and word cloud, among others. CRSs such as Socrative, Yammer [16], and Kahoot [12,34] have been used in health sciences.

Gamification has been also widely employed in a variety of healthcare courses: psychiatric [35], COPD (acronym of Chronic Obstructive Pulmonary Disease) treatment [36], oncology [37], obstetrics [38], urology [39], surgery [40,41,42,43,44] emergency medicine [45], physiology [46,47,48], gynecology [49], internal medicine [50], resuscitation principles [51], anatomy [48,52], urine catheterization [53], radiology [54], and pediatrics [55]. The most used gamification elements in healthcare are scoring [37,38,39,41,42,45,46,50,52,53,55,56,57,58,59,60,61,62,63,64,65,66] and competition [37,38,39,41,42,44,49,50,52,57,58,59,64,65,66,67,68,69]. Rewards [36,41,43,47,48,54,67,68,69], signposting [36,62], and time [45,47,53,54,60,61,62,68] are also frequently used. Other gamification elements less employed in the teaching of health sciences are puzzles [35,70], role playing [35,61,71,72], achievements [73], missions [73], avatars [36,47], levels [36], quizzes [36,73], badges [50,56], levelling [45,56,62,63], quests [56,65], awards [40,74], teams [59,60,61,67], mystery characters [51,60,68], progress [44], social networks [44,58], and storytellings [65]. Certainly, game elements motivate and attract user in teaching activities [50,52,53,56]. All of them aim at ensuring user commitment to perform the learning activities. Evidence on the impact of the gamification on student academic outcomes has been reported in a meta-analysis of 24 empirical studies, involving a total of 3202 participants [75].

## 3. Materials and Methods

Three experiments conducted to assess the educational effectiveness of four gamification elements (ranking, badges, teams, and points) were designed. Two experiments employed a gamified CRS and one experiment employed a non-gamified CRS. In the following subsections the methodology is presented.

### 3.1. Participants and Data Collection

The participants were enrolled in a first-year medical course named General Anatomy of Human Musculoskeletal System (GAHMS) at the University of Murcia. This course is taught during the first 15 weeks of the academic year. GAHMS introduces human anatomy, especially the bone, joint, and muscle systems. A total of three thematic blocks are addressed in the aforementioned course: Unit 1: Description of gross anatomy and introduction to the musculoskeletal anatomy of the pelvis, abdomen, and thorax; Unit 2: Overview of the musculoskeletal anatomy, including both lower and upper limbs; Unit 3: Introduction of the musculoskeletal anatomy, presenting both head and neck composition. GAHMS is a six ECTS (European Credit Transfer and Accumulation System) credit course organized into lectures of four hours per week and skills practice on human cadaveric dissection of 2 h per week to encounter each of the structures of the human body. Students could opt out the study at any time without detriment to their final marks. The participants in the experiment were not repeaters. Moreover, they all had the same background. Therefore, they were all in the same condition to perform the experiment. None of the participants dropped out of the experiment.

The recruitment process started with a verbal presentation and the delivery of a document describing the goal, the procedures, and the tools used in the study. It is worth noting this study passed the approval of the Ethics Committee of University of Murcia.

### 3.2. Instruments

The G-SIDRA (Gamified Immediate Audience Response System in Spanish) is an evolution of an audience response system (https://docentis.inf.um.es/sidra/) called SIDRA (Immediate Audience Response System in Spanish) to endow this tool with gamification elements [76]. In 2018, R-G-SIDRA (gamified SIDRA with ranking) was built by adding three gamification elements (ranking, teams, and points). This extension was used in the academic course 2018/2019. The gamification process was organized in a total of four level stages [77,78]: (1) Business Modeling and Requirements to evaluate the tool and business goals that are documented; (2) Propose the gamification design; (3) Implementation of the software artifacts based on step 2 and test its functionally, and (4) Monitoring and Adaptation to measure business goal achievement and carry out subsequent design modifications if needed. In phase 2, Gamicards were used in the design process to support the gamification [79]. The gamification elements ranking, teams, and points were used to motivate two of the three most common user types (Socializer, and achiever) [80]. The user types Hexad model scale was employed with this aim [81]. In phase 3, a self-built solution in order to support gamification strategies was adopted for the sake of adaption flexibility and to have the control of the whole gamification engine. As reported in [82], self-built solutions to monitor the systems are preferred by experts rather than general gamification platforms.

In 2019, a non-digital gamification element was adopted to promote the gamification process. These elements consisted of metal badges representing gold, silver, and bronze medals, which were delivered at the end of each MCQ test. This system is identified as RB-G-SIDRA (gamified SIDRA with ranking and badges) and was employed in the academic course 2019/2020.

**Figure 1:**
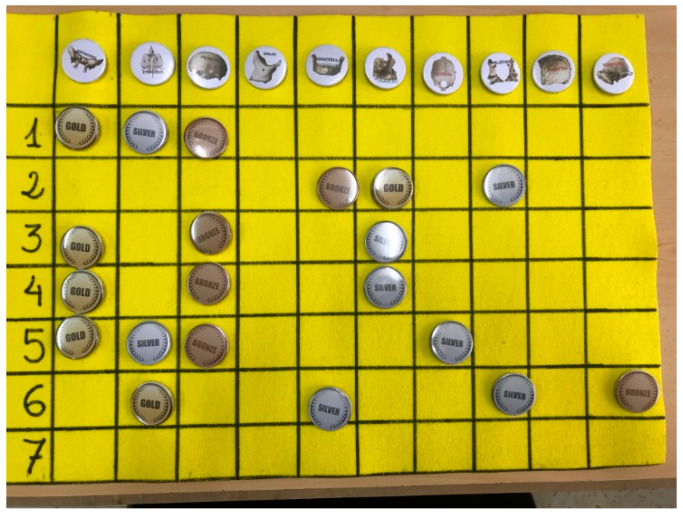
Badges and Team Ranking of RB-G-SIDRA (gamified SIDRA with ranking and badges).

Table 1 shows the game elements used in each SIDRA system. Figure 1 shows the board and the badges used in the RB-G-SIDRA system. Observe that the rows denote the MCQs and the columns represent the teams.

**Figure 2:**
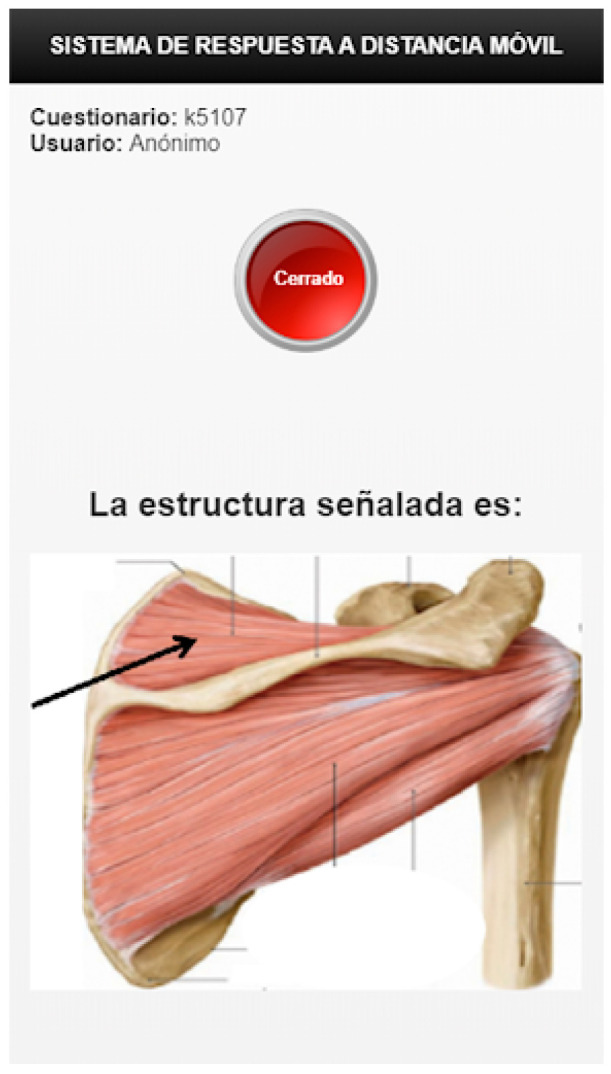
Example of Question Formulated in the G-SIDRA (Gamified Immediate Audience Response System) Mobile Interface.

**Figure 3:**
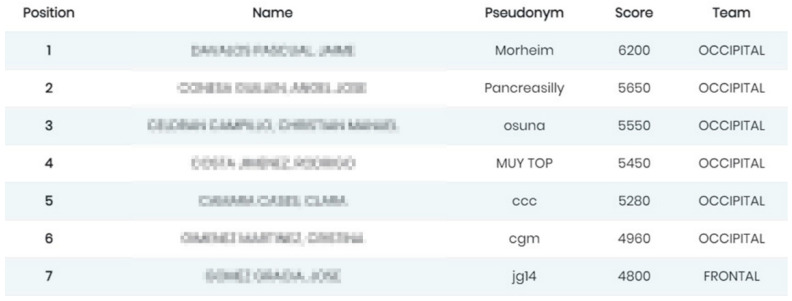
Individual Ranking of A Test.

**Figure 4:**
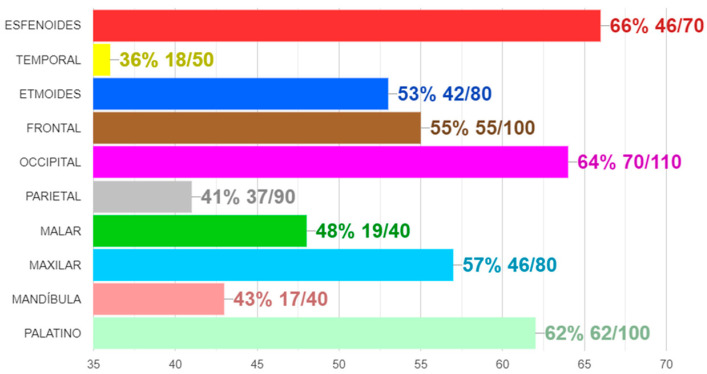
Score and Success Rate of A Test by Team.

In the evolved system, a test is formed by a list of MCQs about a specific topic. The client-server architecture of the SIDRA system provides the instructor with the possibility to gather and evaluate answers to MCQ sent from any device connected to the Internet. A professor can also add respondents, build and launch an MCQ test, download the test results, and display the students’ responses along with a ranking of groups or individuals. Access is granted to professors by sending a G-SIDRA account request to the administrator. A respondent can check the MCQs, complete the questionnaire, and see the percentage of correct answers for each question. All of these actions can be done online during the lecture via web or a mobile app. Figure 2 depicts the mobile interface of G-SIDRA. This interface is common to all gamified SIDRA extensions. Figure 3 and Figure 4 illustrate the gamification elements used in R-G-SIDRA and RB-G-SIDRA: individual ranking, badges, points, and classification of 10 teams, which can be viewed at the end of each test.

### 3.3. Design

Three versions of SIDRA were implemented for comparison in the context of the anatomy of the locomotor system. The sample was split into three groups. A group of 90 students used RB-G-SIDRA in the academic year 2019–2020, another group of 90 participants employed R-G-SIDRA in the academic year 2018–2019 and in the academic year 2017–2018, another group comprising 92 students used SIDRA. The same professors taught and the same explaining method was carried out in the three groups. Moreover, similar training was given concerning GAHMS skills and competences.

Data corresponding to answers of the students from seven, four, and seven MCQ tests taken in the academic years 2017/2018, 2018/2019, and 2019/2020, respectively, were collected. The questions dealt with gross anatomy and musculoskeletal anatomy. Moreover, the students responded to a questionnaire, scoring each question on a five-point Likert scale. The aim was to know the experience on using SIDRA, R-G-SIDRA, and RB-G-SIDRA.

Up-to-date literature on present recommended medical practices was considered when proposing the questionnaire. Furthermore, MCQ-writing recommendations were taken into account [83]. All of the questionnaires consisted of a set of 10 to 14 questions thus avoiding the fatigue effect.

### 3.4. Hypotheses

The following hypotheses were investigated in order to assess the impact on the learning process of students through the use of the aforementioned CRSs. Table 2 depicts a summary of the statistical treatments carried out in this study.

H1. Students using RB-G-SIDRA will obtain higher final exam grades compared to students who used R-G-SIDRA and SIDRA. EducationalTool was the independent variable, with three values: RB-G-SIDRA (academic course 2019/2020), R-G-SIDRA (academic course 2018/2019) and SIDRA (academic course 2017/2018). A dependent variable (Performance, measured using final exam grades) was defined to test the statistical hypothesis.

H2. The students using RB-G-SIDRA will obtain higher MCQ scores than the ones who used R-G-SIDRA and SIDRA. Again, EducationalTool was the independent variable, with three values: RB-G-SIDRA (academic course 2019/2020), R-G-SIDRA (academic course 2018/2019) and SIDRA (academic course 2017/2018). The dependent variable was Score. With this variable the number of correct answers in four MCQ tests was measured. The resulting averages were normalized on 10.

H3. The students with higher MCQ scores will achieve higher final exam grades. A grouping variable called SIDRAScore was used as the independent variable, which gave the low scores (between 0 and first tertile) a value of “1”, the medium scores (between first tertile and second tertile) a value of “2” and the high scores (between second tertile and 10) a value of “3”. Mark in the final exam was entered under the variable name Performance (the dependent variable). The relation between these variables was studied in the three academic courses.

H4. The gamification element individual ranking had an encouraging effect on the students. Ranking variations between each two consecutive tests were calculated for each student, thus resulting in three (VR1_18_19, VR2_18_19, VR3_18_19) and six (VR1_19_20, VR2_19_20, VR3_19_20, VR4_19_20, VR5_19_20, VR6_19_20) variables for the academic course 2018/2019 and 2019/2020, respectively. For example, if a student is ranked on third position in test 1 and on first position in test 2, a variation of two is stored in variable VR1_18_19 in academic course 2018/2019.

H5. The results of the team had an encouraging effect on the results of the individuals. Two variables were used: TeamScoreTx with the average of the team to which the student belongs in test Tx and IndividualScoreTx, which is the MCQ score of the students in test Tx.

H6. Students’ satisfaction with RB-G-SIDRA, R-G-SIDRA, and SIDRA. A questionnaire to know the students’ perspectives concerning their experience with SIDRA systems was completed by the participants in the experiments. A five-point Likert-type scale (5 = very high; 4 = high; 3 = medium; 2 = low; 1 = very low) was used in a nine-question questionnaire with also a Yes/No question.

### 3.5. Statistical Analysis

The tools SPSS 24.0 (IBM Corporation, Armonk, NY, USA) and Office Excel 2020 (Microsoft Corporation, Redmond, WA, USA) allowed to analyze the data and generate the figures. In order to detect statistically significant differences, a conventional significance level of 0.05 was used. The Kolmogorov–Smirnov statistical test allowed to verify if the study groups followed a normal distribution. When data of the dependent variable was not normally distributed, non-parametric tests were used. Particularly, the Mann–Whitney U test allowed to compare differences between the medians of two independent groups. Moreover, the Kruskal–Wallis H test or the one-way ANOVA on ranks was performed between the medians of more of two independent groups to compare the differences. Spearman’s correlation was also employed, allowing to measure the direction of association and the strength between two variables representing paired observations which are not normally distributed.

## 4. Results

H1. The performance (final exam score) varies as regards academic course, with Kruskal–Wallis χ2(2) = 14.349, *p* = 0.001. The highest average score was obtained by students using RB-G-SIDRA in the academic course 2019/2020 (M = 7.44; SD = 1.33) and the lowest average score by students using SIDRA in the academic course 2017/2018 (M = 6.43; SD = 1.78). Post-hoc paired comparisons were applied by using Mann–Whitney U tests (non-parametric). Statistically significant differences were found between RB-G-SIDRA and SIDRA, U = 39.211 adjusted *p* = 0.001 and RB-G-SIDRA and R-G-SIDRA U = 31.157 adjusted *p* = 0.015.

H2. The MCQ score varies as regards academic course, with Kruskal–Wallis chi-squared (2) = 96.217, *p* < 0.001. The highest average score was obtained by students using RB-G-SIDRA in the academic course 2019/2020 (M = 6.67; SD = 1.11) and the lowest average score by students using SIDRA in the academic course 2017/2018 (M = 3.98; SD = 1.42). Post-hoc paired comparisons were applied by using Mann–Whitney U tests (non-parametric). Statistically significant differences were found between RB-G-SIDRA and SIDRA, U = −90.521 adjusted *p* < 0.001, and between R-G-SIDRA and SIDRA, U = −87.998 adjusted *p* < 0.001. However, statistically significant differences were not found between RB-G-SIDRA and R-G-SIDRA U = −2.523 adjusted *p* = 1.

H3 Table 3 shows the average final exam score for each group formed by tertiles in SIDRA, R-G-SIDRA, and RB-G-SIDRA.

Academic course 2017–2018. There was a statistically significant difference between groups as determined by one-way ANOVA (F(2.71) = 11.243, *p* < 0.001). A Tukey post hoc test revealed that the final exam mark was statistically significantly higher in the group of students with high score (7.70 ± 1.22 points) in SIDRA compared to the group of students with medium score (6.59 ± 1.29 points, *p* = 0.030) and low score (5.71 ± 1.85 points, *p* < 0.001) in SIDRA. There was no statistically significant difference between the group of students with medium score compared with low score (*p* = 0.101).

Academic course 2018–2019. There was a statistically significant difference between groups as determined by one-way ANOVA (F(2.64) = 15.096, *p* < 0.001). A Tukey post hoc test revealed that the final exam mark was statistically significantly lower in the group of students with low score (5.23 ± 2.02 points) in R-G-SIDRA compared to the group of students with medium score (6.96 ± 1.23 points, *p* = 0.001) and high score (7.73 ± 1.28 points, *p* < 0.001) in R-G-SIDRA. There was no statistically significant difference between the group of students with medium score compared with high score (*p* = 0.226).

Academic course 2019–2020. There was not a statistically significant difference between groups as determined by the Kruskal–Wallis H test χ2(2) = 4.042, *p* = 0.133.

**Figure 5:**
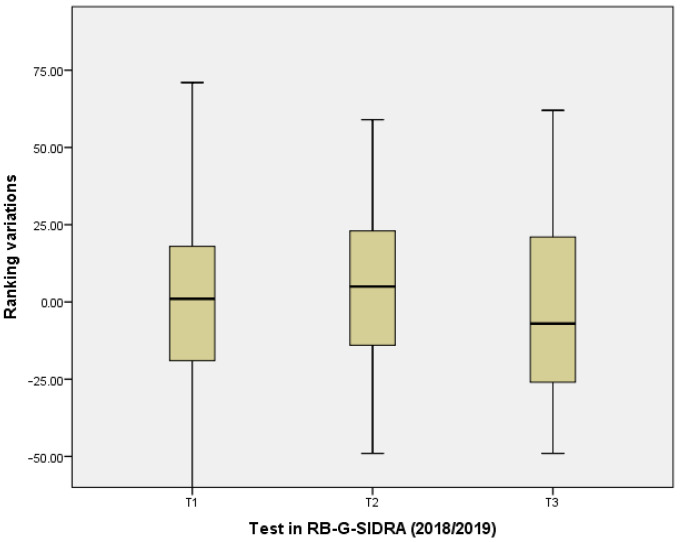
Box Diagram for Ranking Variations in the Academic Course 2018/2019 R-G-SIDRA (gamified SIDRA with ranking).

**Figure 6:**
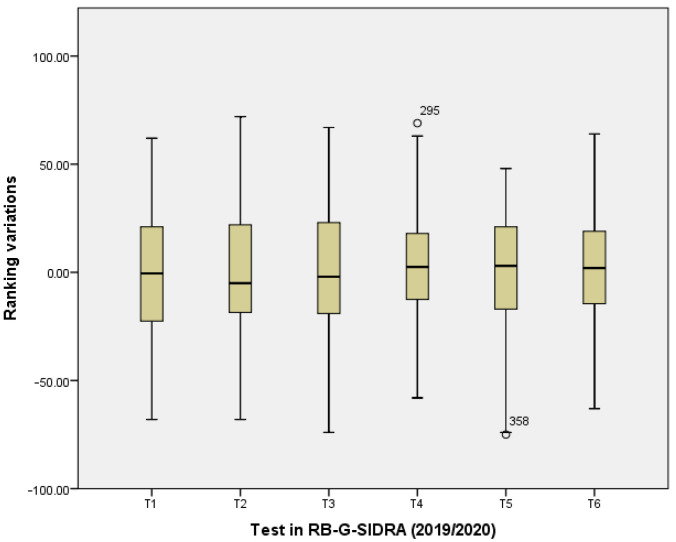
Box Diagram for Ranking Variations in the Academic Course 2019/2020 (RB-G-SIDRA).

H4. Ranking variations between each two consecutive tests were calculated for each student in RB-G-SIDRA and R-G-SIDRA, which included the gamification element ranking. Figure 5 and Figure 6 show two box diagrams to study the dispersion of data. The dispersion of the ranking variations revealed a slight decreasing trend as the tests are taken during the academic year. This means that the classification shows some tendency to stabilize. Notice that R-G-SIDRA diagram (academic course 2018/2019) satisfies that, in the last test, more than half of the students achieved negative ranking variations. In contrast, RB-G-SIDRA diagram (academic course 2019/2020) satisfies that, in the last test, some students obtained remarkable increases concerning ranking variations (first quartile).

H5. Spearman’s rank correlation coefficients between individual and team scores for each MCQ test in RB-G-SIDRA and R-G-SIDRA were calculated. Significant correlations between individual and team scores were found in all of the tests as shown in Table 4. Notice that the correlations become stronger as the tests progress. These findings revealed that the inertia of the team can have a crucial influence on the individual performance of each team member.

H6. Table 5 presents several statistical parameters such as the means, standard deviations and medians of the scores obtained for 87, 71, and 38 students who used RB-G-SIDRA, R-G-SIDRA, and SIDRA, respectively. The use of the three SIDRA systems was positively evaluated by the students, with median 4 or 5 in all of the questions for the three systems, confirming hypothesis H6. Moreover, the gamification elements used in the learning of human anatomy (ranking, badges, teams and points) were positively evaluated as a motivational factor in the classroom (median 4 in Q6). The system allows trainees to understand better theoretical and practical concepts at the same time (median 4 or 5 in Q3 in the three systems). Teamwork also was highly valued (median 5 and 4 in Q7 in R-G-SIDRA and RB-G-SIDRA, respectively). Significant differences are also found in the assessment of the climate in class (1 point difference in medians in Q8).

Finally, there was a question with a dichotomous answer asking if you would use the SIDRA system in other courses. Observe that 96%, 100%, and 99% of the students (using RB-G-SIDRA, R-G-SIDRA, and SIDRA, respectively) would like the system to be used in more subjects.

## 5. Discussion

In this section, the main findings on hypotheses investigated to assess the impact of the use of gamified and non-gamified CRSs on the learning process of students are examined, analyzed, and compared with those of other studies.

### 5.1. Improving learning Outcomes

H1 hypothesis testing revealed that in the final exam of the anatomy course, the marks of the students who used RB-G-SIDRA were significantly better than those of the SIDRA group. These results confirmed previous research in which the use of gamified CRSs was studied [50,52,84]. Increased knowledge has been reported by a high number of experiments [39,40,43,47,50,51,52,55,56,58,63,64,65,67,68,69]. It is observed that the positive effect on students’ knowledge is independent of age and gender [85]. Gamification has been widely used in healthcare education [86].

In particular, an experiment to study the impact of points and leaderboard in computer science and psychology education reported a statistically significant increase on users’ performance [87], which provides indications to believe that the gamification elements adopted in RB-G-SIDRA are effective. In contrast, there were no statistically significant differences when investigating the ranking event in our experiment (H1), that is to say, there were no statistically significant differences between R-G-SIDRA and SIDRA.

Gamification has also been successfully implemented in human anatomy education [87,88]. The highest post-test versus pre-test scores were found in a group that adopted a gamified approach, being different from the non-gamified approach used in the other two groups [89]. Nevertheless, an experiment on leaderboard and badges revealed negative effects with the marks of the students’ final exams attending to a communication course [90]. Notice that ranking can generate both stress by the competition and feelings of inferiority in students, resulting in a reduced sense of autonomy and competence [91], thus negatively impacting the performance of the student. Those who fail to go up in the ranking table may feel a lower competence, which could lead to discouragement [92]. Therefore, lower-performing students may not benefit from the gamified presentation [93]. That was the case in our experiment (H1) as previous mentioned, since R-G-SIDRA did not improve the student performance compared to SIDRA. To remove this limitation, R-G-SIDRA depicted leaderboard only when each test was finished. Moreover, the scores were removed when starting each test.

Our study found significant differences between SIDRA and RB-G-SIDRA. This fact leads us to conclude that badges have a positive influence on learning outcomes. Previous research [50,56] revealed that students who received badges are more likely to achieve better marks. To avoid the comparative progress tracking provided by leaderboards/rankings, badges are excellent alternatives as game mechanics. These gamification elements allow instructors to show failure to the student without imposing punishment [94]. Moreover, badges reinforce certain learning behaviors such as perseverance. Notice that scientific evidence supports the use of a dopamine reward system as a powerful physiologic ally to achieve effective learning. Dopamine, which produces satisfaction, is released each time the student responds correctly and receives a badge [95]. Students strive to increase mastery of course content with the ultimate goal of maintaining the flow of satisfaction. Flow occurs when students are engaged in an activity (physical, mental, or both) in such a way that they lose track of time and the outside world [96]. After initial excitement at earning badges, students can be less motivating than the leaderboard [50] when they lost interest over time. For this reason, this flow must be considered by design [56] and gamification must be planned to keep students continuously satisfied. Any additional classroom tasks such as textbook reading and professor handouts must be integrated in the gamification activities to minimize the interruption of flow [97]. Our proposal addresses this point in the gamification process followed to keep students continuously satisfied.

The results obtained show no statistically significant difference between groups formed by tertile based on the RB-G-SIDRA score. Final exam average and SIDRA system score intervals are significantly higher in the three groups formed for RB-G-SIDRA with respect to the groups formed by R-G-SIDRA and SIDRA, as observed in Table 3. As an example, the score of the third tertile interval in RB-G-SIDRA (8.7 ≤ SCORE ≤ 10) with M = 7.989 is higher than that in R-G-SIDRA (8 ≤ SCORE ≤ 10) with M = 7.733 and SIDRA (6.8 ≤ SCORE ≤ 10) with M = 7.702. The same thing happened in the rest of tertile score intervals. We can conclude that badges included in RB-G-SIDRA allow students to achieve better and more homogeneous learning outcome during the course. This finding is confirmed by previous research [56].

### 5.2. Effect of Rankings and Teams

Social Comparison Theory (SCT) affirms that each individual possesses an inherent drive to receive accurate self-evaluations with the aim of ascertaining the validity of their own opinions and judgments [98]. Previous research has been reported on importance of the role played by social comparison in the development of academic performance [99]. Academic competition allows instructors to underpin a learning environment with social comparison. Notice that 57 out of 90 students achieved a higher or equal number of positive ranking variations than negative ranking variations in the academic course 2019/2020. This is an indicator of the motivation behind the competition. In contrast, 33 out of 90 students had a higher number of negative ranking variations than positive ranking variations. This group of students may be frustrated and have feelings of incompetence and dependency [91], thus falling into a cycle of disinterest in the subject [92]. These students obtained lower performance with an average score in the final exam of 7.14, which is lower than average score of the whole group (M = 7.44). They do not benefit from the gamified activities as confirmed in previous research [93]. Obviously, the motivating factor of competition may vary depending on many factors such as ethnicity, society, age, and individual preferences in the learning styles [64,100]. This duality present in the competition with respect to student motivation has been confirmed in other experiments [37]. Finally, regarding the ranking variations, similar conclusions to those of academic course 2019/2020 were found in the academic course 2018/2019.

Part of the activities carried out by health professionals involve working in teams in different clinical environments [61]. Therefore, learning and understanding the dynamics of teamwork is an added value provided by the gamification element team. For example, questions are formulated to allow students to explore and discuss aspects of theory and practice in a range of common situations in a hospital. The benefit is mutual among team members as evidenced by the positive correlation between team ratings and the individual ratings of each team member in our study as confirmed in H5. In the learning environment proposed in R-G-SIDRA and RB-G-SIDRA gamified SIDRA, team competition was adopted by using one device per student as it is the preferred modality for students [60]. Observe that CRS promotes social cohesion in classrooms through viewing responses sent by peers over time or knowing what classmates think [60]. The data generated by CRSs can be used to spark discussion [101] and to develop communication skills to learn from and with each other. In addition to being enjoyable [59], teams allow instructors to foster the idea of social fabric since students build a higher level of confidence and have a greater willingness to collaborate after playing games together [102]. Competition by teams also endowed SIDRA with an educational instrument that allowed a balance between cooperation and competition [64].

### 5.3. Survey

Satisfaction in using gamification has been widely recognized in previous studies on health professions education [46,52,55,73], in general, and using gamified CRS [12], in particular. This is confirmed in our survey in which students highly rated the use of the system in the classroom (question Q1 in academic courses 2018/19 and 2019/20).

Our survey showed that the gamified systems were more motivating than the non-gamified system in students’ learning process (question Q2). This finding is confirmed in a previous experiment. Significant differences were found on the motivation of students who took lectures with a gamified CRS and those who took lectures with a non-gamified CRS [103]. In most of the educational innovations, students are very enthusiastic at the beginning when using a CRS for the first time. However, novelty and its benefits are lost after being used several times [104].

Notice that the evaluation of the feedback provided by the instructor (RB-G-SIDRA and R-G-SIDRA) is notably superior to that of SIDRA (two-point difference in medians in Q4). The same feedback was given by the same instructors in the three systems. Probably, the students highly valued the discussion groups created in RB-G-SIDRA and R-G-SIDRA.

In a survey responded by students enrolled on an undergraduate human anatomy course, 50% of participants felt that the competitive situation motivated them, whereas 25% of participants did not agree [52]. In our survey (question Q6) the results are varied according to whether or not badges are used: RB-G-SIDRA (M = 4.34) and R-G-SIDRA (M = 3.86).

Our survey (question Q7) achieved similar results to a previous study based on a simulation game, in which 94% of participants considered that teamwork was important for their nursing learning activities [61]. An educational ultrasound event named Sound Games was also used for medical training in emergency medicine [59]. Most of the participants (93.75%) agreed or strongly agreed that working in a team was enjoyable. Health disciplines can benefit from this game element to understand the dynamics of many clinical environments. Finally, our survey revealed intentions to continue the use of gamification elements in other subjects in similar percentages to other surveys in the pediatric primary care (100%) [67] and blood grouping (98%) [73].

Fun is another benefit reported on literature [35,59,61,73]. Q8 shows that classes were more dynamic and fun when using RB-G-SIDRA (M = 4.66) and R-G-SIDRA (M = 4.77). The results were slightly lower in the non-gamified system. This is in line with a study in which 99% students indicated fun in using an online blood grouping game [73].

Notice that the field of study can influence the perception of students on the use of gamified CRS. Students in technological disciplines can perceive CRS as a more useful tool than student in social science disciplines [103].

## 6. Conclusions

This paper reported the effects of three experiences, two with a gamified CRS and one CRS without gamified features, on student performance and perceptions in a course on anatomy of the locomotor system. Findings supported that the use of ranking, badges, teams, and points in a CRS had a positive statistically significant effect on the marking of the students’ final exam. Strong evidence was found considering the benefit of the badges in RB-G-SIDRA in comparing R-G-SIDRA. Moreover, statistical tests revealed that the activity of the team can have an important impact on the individual performance of each team member. Perceptions collected in a survey about gamification confirmed higher motivation to participate in the classroom using RB-G-SIDRA with respect to R-G-SIDRA.

The improvement in the learning outcomes of the course could be summarized basically in that the students were able to identify more easily the axes and planes of orientation and their relationship with the most important anatomical structures, as well as the topographical regions of interest. In addition, they were able to adequately use anatomical terminology with respect to the morphology and global structure of the human body, especially with respect to the bones, muscles, and joints of the human body, acquiring these concepts more easily. The academic results showed that the use of RB-G-SIDRA led to an improvement in the acquisition of the learning objectives.

In comparing, rankings and badges, this last gamification element allows instructors to reward students without the stress and the possible feelings of inferiority produced by the competition. For students who are lagging behind, rankings can negatively impact on their performance. However, badges provide instructors with an excellent resource to show failure to the student without infringing a penalty such as being at the bottom of a ranking. Our results confirmed the evidence found in most of the scientific literature on the effects of gamification on health science student academic performance, motivation, and engagement. New experiments should be designed to compare the impact of the different gamification elements, in consideration of the types of learners and players. As a result, a gamified adaptive learning system could be built to address the different types of learning.

The integration of gamification elements into a CRS is a feasible settlement to tackle overcrowded classrooms, which prevent adequate communication with students. Moreover, these systems enable safe and sustainable education to face the new reality caused by COVID-19 [105]. In the synchronous education, a gamified CRS can be used in live interactive lessons by videocalls, whereby instructors and students are able to interact in real-time. A gamified CRS satisfying educational standards such as IMS (acronym of “Instructional Management System”) Content Packaging and SCORM (acronym of “Shareable Content Object Reference Model”) specifications can be integrated into Learning Management Systems (LMS) such as Sakai or Moodle, which are widely used in educational center. The visits to the academic organization can be drastically reduced when the learner attendance is not required. In future work, we intend to integrate G-SIDRA into an LMS such as Sakai in order to facilitate the adoption of this type of environment.

## Figures and Tables

**Table 1:** Gamification Elements in SIDRA Systems (Immediate Audience Response System in Spanish). R-G-SIDRA (gamified SIDRA with ranking); RB-G-SIDRA (gamified SIDRA with ranking and badges).

	COURSE	RANKING	BADGES	TEAM	POINTS	Nº OF MCQ TEST
SIDRA	2017/18	NO	NO	NO	NO	7
R-G-SIDRA	2018/19	YES	NO	YES	YES	4
RB-G-SIDRA	2019/20	YES	YES	YES	YES	7

**Table 2:** A Summary of the Statistical Treatments Performed for Each Hypothesis.

	H1	H2	H3	H5
Test	Kruskal–Wallis	Kruskal–Wallis	ANOVA, Tukey post hoc test and Kruskal–Wallis	Spearman’s correlation
Independent variable	SIDRA system used	SIDRA system used	Total correct answers in SIDRA	Individual ScoreTX
Dependent variable	Final marks	Total correct answers in SIDRA	Final marks	TeamScoreTx

**Table 3:** Descriptive Statistics for Final Exam. “N”: Number of students; “M”: Mean; “SD”: Standard deviation.

Academic Year 2017/2018—Final Exam Score			
	N	M	SD
SIDRA SCORE. FIRST TERTILE (0 ≤ SCORE < 5)	25	5.710	1.853
SIDRA SCORE. SECOND TERTILE (5 ≤ SCORE < 6.8)	24	6.596	1.297
SIDRA SCORE. THIRD TERTILE (6.8 ≤ SCORE ≤ 10)	25	7.702	1.223
Academic Year 2018/2019—Final Exam Score			
	**N**	**M**	**SD**
R-G-SIDRA SCORE. FIRST TERTILE (0 ≤ SCORE < 6.35)	22	5.235	2.026
R-G-SIDRA SCORE. SECOND TERTILE (6.35 ≤ SCORE < 8)	22	6.960	1.238
R-G-SIDRA SCORE. THIRD TERTILE (8 ≤ SCORE ≤ 10)	23	7.733	1.289
Academic Year 2019/2020—Final Exam Score			
	**N**	**M**	**SD**
RB-G-SIDRA SCORE. FIRST TERTILE (0 ≤ SCORE < 7.6)	27	6.803	1.651
RB-G-SIDRA SCORE. SECOND TERTILE (7.6 ≤ SCORE < 8.7)	26	7.443	1.056
RB-G-SIDRA SCORE. THIRD TERTILE (8.7 ≤ SCORE ≤ 10)	27	7.989	1.657

**Table 4:** Spearman’s Rank Correlation Coefficient Results between Individual and Team Score in RB-G-SIDRA (gamified SIDRA with ranking and badges) and R-G-SIDRA (gamified SIDRA with ranking). “Tx”: Test x; “CC”: Correlation Coefficient; “N”: Sample Size; “*p*”: *p* Value.

Academic Year 2018/2019																				
	T1				T2						T3						T4			
CC	0.589				0.564						0.390						0.829			
N	78				78						78						78			
*p*	0.000				0.000						0.000						0.000			
Academic Year 2019/2020																				
	T1		T2				T3		T4				T5		T6				T7	
CC	0.468		0.701				0.624		0.607				0.729		0.722				0.660	
N	87		87				87		87				87		87				87	
*p*	0.000		0.000				0.000		0.000				0.000		0.000				0.000	

**Table 5:** Students’ Perceptions. “M”: Mean; “SD”: Standard Deviations; “Md”: Median.

Id	Question	SIDRA 2017/18			R-G-SIDRA 2018/19			RB-G-SIDRA 2019/20		
		M	SD	Md	M	SD	Md	M	SD	Md
Q1	Are you pleased with the use of the system in the classroom?	4.29	0.65	4	4.73	0.53	5	4.55	0.61	5
Q2	Does the system motivate you in your learning process?	4.37	0.69	4	4.61	0.64	5	4.41	0.77	5
Q3	Does the system helped you to better understand both theoretical and practical concepts?	4.24	0.65	4	4.39	0.73	5	4.09	0.92	4
Q4	Does the instructor’s feedback help you in your learning process?	3.53	1.20	3	4.67	0.53	5	4.42	0.90	5
Q5	Is the time spent on the system based learning activity appropriate?	4.63	0.69	5	4.39	0.76	5	4.15	0.92	4
Q6	Do the gamification elements included in the system motivate participation in the classroom?	-	-	-	3.86	1.13	4	4.34	1.01	5
Q7	Does teamwork helped you to improve in your learning process?	-	-	-	4.38	0.85	5	4.22	0.91	4
Q8	Are classes more dynamic and fun when using the system?	4.32	0.67	4	4.77	0.54	5	4.66	0.66	5
Q9	Your final assessment of the platform is:	4.28	0.72	4	4.61	0.62	5	4.47	0.63	5

## Data Availability

Not applicable.
